# The Correlation Between the Chemical Composition and the Microstructure of the Polysaccharides of Two Varieties of Mexican Red Prickly Pear Fruits

**DOI:** 10.3390/foods13233914

**Published:** 2024-12-04

**Authors:** Yvonne Roman Maldonado, Socorro Josefina Villanueva-Rodríguez, Hilda María Hernández-Hernández, Eduardo Terrés, Jesus Cervantes Martinez

**Affiliations:** 1 Food Technology Unit, Center for Research and Assistance in Technology and Design of the State of Jalisco A.C. (CIATEJ), Guadalajara 44270, JAL, Mexico; yvroman_al@ciatej.edu.mx (Y.R.M.); svillanueva@ciatej.mx (S.J.V.-R.); 2 Institute of Basic Sciences and Engineering, CONAHCYT-Universidad Autónoma del Estado de Hidalgo (UAEH), Pachuca 42184, HGO, Mexico; 3 Laboratory Management, Gustavo A, Madero Mexican Petroleum Institute (IMP), Mexico City 07730, Mexico; eterres@imp.mx; 4 Analytical and Metrological Services Unit, Center for Research and Assistance in Technology and Design of the State of Jalisco A.C. (CIATEJ), Guadalajara 44270, JAL, Mexico; cervantes@ciatej.mx

**Keywords:** fibrillar, interactions, food matrix, neutral sugars, methoxylation, degree of esterification

## Abstract

The red prickly pear fruit (*Opuntia ficus-indica* L. Mill), endemic from Mexico’s semi-desert regions and present in North Africa and Southern Europe, particularly Italy and Spain, is a valuable source of nutrients, bioactive compounds, and polysaccharides. This study used non-destructive techniques like microscopy and Raman and infrared (IR) spectroscopy to characterize polysaccharides extracted from two red prickly pear varieties. The polysaccharides constitute approximately 80% of the peel and 39–18% of the pulp; microscopy provided insights into its microstructural details, while Raman and IR spectroscopy enabled the identification of its specific functional groups. The results revealed distinct microstructural attributes: mucilage displays a microstructure influenced by the ratio of acidic to neutral sugar monomers; pectin exhibits a low degree of methoxylation alongside a characteristic egg-box structure facilitated by calcium ions; hemicellulose presents a delicate, porous layer; and cellulose reveals a layered microstructure supported by thin or robust fibers and calcium crystals. The functional groups identified via Raman and IR spectroscopy provided specific information that could be used to infer chemical interactions influenced by functional groups like hydroxyl, carboxyl, and methyl, suggesting potential binding, stabilization, and water retention properties that enhance their utility as functional ingredients in food products. These findings, obtained using non-destructive methods, enhance the understanding of the compositional and microstructural characteristics of polysaccharides in the red prickly pear, which, in turn, can be used to predict their promising technological applications as functional ingredients.

## 1. Introduction

The red prickly pear (*Opuntia ficus-indica* L. Mill) is a fruit belonging to the cactus family, which is native to Mexico and the most consumed within the *Opuntia* genus. Pre-Hispanic cultures not only consumed it but also used it as a natural dye and for treating various ailments, making it a vital part of Mexican culture. Although Mexico remains the leading global producer of prickly pears fruits, its consumption is currently limited across the country, resulting in significant food waste and missed economic opportunities, as well as potential environmental impacts. This fruit thrives in arid regions with low water requirements and is cultivated in parts of Latin America, Africa, and the Mediterranean, highlighting its potential as a sustainable crop for the future [1].

In contrast, the global demand for the red prickly pear is increasing due to its impressive nutritional composition. Numerous studies indicate that it serves as a valuable source of nutrients and bioactive compounds, including betalains (natural pigments), polysaccharides such as soluble dietary fibers (mucilage and pectin), and insoluble fibers (cellulose and hemicellulose). Polysaccharides are important biomacromolecules with numerous beneficial functions and various industrial applications. Recent research has highlighted their numerous health benefits, particularly their role in modifying a food matrix’s structure and influencing glycemic index control [2,3]. The biological, prebiotic, and technological properties of polysaccharides are of significant interest due to their potential contributions to food formulations from nutritional and functional perspectives. Studies in this area have shown that the functions and properties of polysaccharides are closely linked to their structural features [4]. Their structure provides visual information that reflects their molecular arrangement, which is influenced by the chemical composition of the molecules, while also confirming their functional groups identified via spectroscopy. As such, structural information is fundamental to understanding the relationship between polysaccharide structure and function. Compared to other biological macromolecules, polysaccharides exhibit significantly greater structural complexity due to their diversity of monomeric units and linkage types [4,5]. This complexity, which ranges from simple linear chains to branched, intricate structures, directly influences their physical characteristics and, consequently, their functional properties [6].

Furthermore, the interactions between polysaccharides and a food matrix are influenced by various factors, such as their physicochemical properties related to their morphology (surface area, shape, and porosity), chemical composition (sugar content and solubility), and molecular architecture (molecular weight, degree of esterification, and functional groups) [2]. Hence, polysaccharide structural analysis is an important but challenging task.

Different analytical techniques, including structural, spectral, chemical, and chromatographic analysis, are applied to elucidate the fine chemical structure of polysaccharides. There are many methods of spectroscopic analysis that can be used to determine the molecular structure of polysaccharides. These methods include vibrational spectroscopy techniques, which are powerful probes of molecular structure, and their advantages for biomedical research (with a special emphasis on proteins and nucleic acids) are widely recognized in the literature [7]. Infrared (IR) spectroscopy is a rapid, non-destructive, and accessible technique widely used for polysaccharide structural analysis. Numerous studies highlight its applications, including detailed spectral features of various carbohydrates [8,9] and its use in analyzing cellulose in isolated form and within plant cell walls [10]. Additionally, IR imaging techniques used for characterizing polysaccharides in plant cell walls have been thoroughly reviewed, demonstrating their versatility in this area [11,12]. Infrared and Raman spectroscopy are complementary techniques for molecular structural analysis. Since they differ in selection rules, bands due to vibrations changing molecular polarizability are easily detectable in Raman spectra. Conversely, motions, which change dipole moments, can be better recognized using IR spectroscopy [9].

Furthermore, it is important to highlight that for an optimized utilization or design of materials or ingredients, basically, four elements must be understood and controlled as the following: the structural and chemical compositions, the synthesis or obtention and processing, the properties, and the performance of a material [11].

Therefore, this study aims to correlate the microstructural characteristics obtained by microscopy with the chemical composition of polysaccharides in the prickly pear fruit, comparing those in the pulp and peel. Using specific, rapid, and non-destructive techniques—Raman, IR spectroscopy, and microscopy—this study provides detailed insights into their structural features, which are closely linked to their functional and technological properties. This foundational understanding serves as a basis for the further exploration of their potential applications.

## 2. Materials and Methods

### 2.1. Raw Materials

Two varieties of the red prickly pear from Zacatecas, Mexico, belonging to the *Opuntia* genus, were studied as the following: “Cardona” (*Opuntia streptacantha*) and “Zacatecas” (*Opuntia* spp.). Cardona fruits were sourced from a local market in Zacatecas, while Zacatecas fruits were obtained from a market in the state, Jalisco. All selected fruits were at commercial ripeness and free from mechanical or microbiological damage. They were washed with water and soap and then stored at 5 °C until polysaccharide extraction.

### 2.2. Polysaccharide Extraction

The polysaccharides from the peel (pe) and the pulp (pu) of the two varieties of red prickly pear were extracted through a sequential extraction process: (i) mucilage; (ii) pectin; (iii) hemicellulose; and (iv) cellulose. The process followed previous reports [3] with slight modifications, included extending the agitation time to 6 h for hemicellulose extraction and reducing to only a 5% KOH concentration.

#### 2.2.1. Mucilage Extraction

Mucilage extraction was performed using 2 g of fresh red prickly pear pulp or peel from each variety. A total of 10 mL of distilled water was added, and the mixture was brought to a boil (90 °C) in a water bath for 30 min. Afterward, it was centrifuged at 1400 × *g* for 5 min to separate the solid phase from the liquid phase. The supernatant containing the mucilage was collected, which was precipitate 1, and stored to extract other polysaccharides. Then, 20 mL of ethanol at −20 °C was added to the supernatant, and it was maintained at 5 °C for 12 h to encourage mucilage precipitation. The ethanol was decanted, and the sample was left at room temperature for 6 h to evaporate any remaining ethanol. The obtained mucilage was prepared for subsequent purification via dialysis.

#### 2.2.2. Pectin Extraction

Pectin extraction involved adding 10 mL of a chelating solution (ammonium oxalate 0.5% *w*/*v*) to precipitate 1 (from Section 2.1). The mixture was then heated to boil (90 °C) in a water bath for 30 min. After boiling, it was centrifuged at 1400 × *g* for 5 min, and the supernatant containing pectin was collected. Precipitate 2 obtained in this step was stored to extract the remaining polysaccharides. Then, 20 mL of ethanol at −20 °C was added to the collected supernatant, and it was maintained at 5 °C for 12 h to stimulate pectin precipitation. Subsequently, the ethanol was decanted, and the sample was left at room temperature for 6 h to evaporate any remaining ethanol. The pectin was prepared for further purification via dialysis.

#### 2.2.3. Hemicellulose and Cellulose Extraction

A hemicellulose fraction was obtained by adding 10 mL of 5% KOH to precipitate 2 (from Section 2.2). It was then continuously agitated for 6 h at room temperature (23 ± 3 °C). The solid phase was separated from the supernatant via centrifugation (1400× *g*, 15 min). The supernatant, which contained hemicellulose, had 20 mL of ethanol added at −20 °C and was kept at 5 °C for 12 h to encourage hemicellulose precipitation. Additionally, 4–5 drops of concentrated HCl were added. The final precipitate obtained from the centrifugation was the cellulose fraction, which was washed with water until it reached a neutral pH.

### 2.3. Polysaccharide Purification

Polysaccharides were purified using membranes to eliminate all non-essential compounds that could be trapped. Each polysaccharide was individually placed in dialysis membranes (molecular weight retention of 14 kDa or more, D9652, Sigma-Aldrich, St. Louis, MO, USA) for final purification. These membranes were then placed in containers with 500 mL of distilled water, which was renewed every 4 h, and kept under constant agitation for 72 h. Subsequently, the polysaccharides were lyophilized, and the results are expressed as % dry weight.

### 2.4. Structural Characterization of Polysaccharides

#### 2.4.1. Raman Spectroscopy

Raman spectroscopic measurements were performed using a spectrometer (NIR-QUEST, Ocean Optics, Oxford, UK) equipped with a germanium detector, coupled to a microscope with a 40× objective. A diode laser emitting at 785 nm with a power of 70 mW and an integration time of 20 s was used as the excitation source. Spectra were collected between 2000 and 200 cm^−1^ using SpectraSuite software, and data analysis was performed using SpectraGryph version 1.2 spectroscopy software (Oberstdorf, Germany).

#### 2.4.2. FT-IR Spectroscopy

The samples were analyzed using an FTIR spectrometer Cary 630 (Agilent Technologies, Santa Clara, CA, USA) with a resolution of 4 cm^−1^ and 10 scans. The samples were placed on the surface of the diamond prism in the attenuated total reflection onfiguration and pressed. The results were collected using MicroLab Agilent Technology software version 5.7 within a range of 650–4000 cm^−1^ for each sample, and data analysis was performed using SpectraGryph version 1.2 software (Oberstdorf, Germany).

#### 2.4.3. Scanning Electron Microscopy (SEM)

Polysaccharides were observed with a scanning electron microscope (XL30 ESEM, Philips, The Netherlands) and an acceleration voltage of 25 kV at a low vacuum (1.2 mBar). Images were taken at magnifications of 300 and 500×. Polysaccharides were mounted on cylindrical aluminum sample holders with double-sided carbon tape.

#### 2.4.4. Statistical and Data Analysis

All values are expressed as the mean ± standard deviation (SD) of three completely independent replicates. Statistical data analysis was performed using one-way analysis of variance (ANOVA). The level of statistical significance was *p* < 0.05.

## 3. Results and Discussion

### 3.1. Total Polysaccharide Content

Table 1 presents the polysaccharide content in the peel and pulp of the red prickly pear. In the peel, polysaccharides constituted about 80% of the dry weight in both studied varieties, while in the pulp, the concentration ranged between 18% (Cardona) and 39% (Zacatecas) (dry weight). Cellulose is the predominant polysaccharide in both the peel and pulp of both varieties, as reported in previous studies [3]. However, the Zacatecas variety pulp contains significantly higher levels of cellulose, pectin, and mucilage than Cardona, supporting previously reported values for the mucilage content in the prickly pear [13]. Hemicellulose is primarily found in the peel of the Cardona variety, while the pectin content is similar in the peels of both varieties.

### 3.2. Structural Characterization of Polysaccharides

The identity of purified polysaccharide fractions from the pulp and peel of red prickly pear varieties Cardona (C) and Zacatecas (Z) was analyzed using IR and/or Raman spectroscopy to identify and confirm the characteristic functional groups of each polysaccharide; this information was then correlated with the microstructures observed using scanning electron microscopy (SEM).

#### 3.2.1. Mucilage

**Figure 1:**
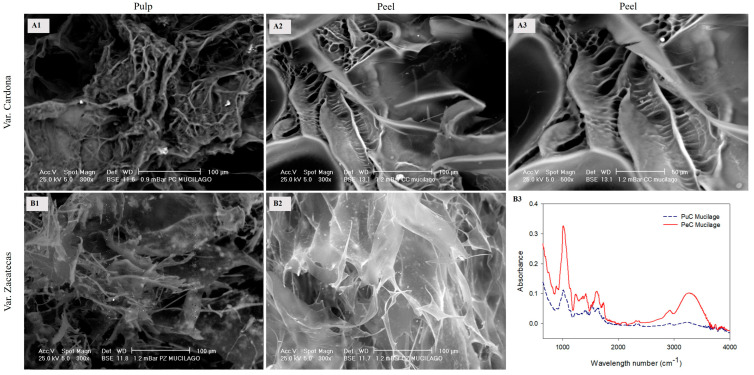
SEM images of mucilage from the peel and pulp obtained from the red prickly pear, Cardona ((**A1**,**A2**)—300×; (**A3**)—500×) and Zacatecas varieties ((**B1**,**B2**)—300×). The IR spectrum of mucilage obtained from the pulp and peel of the Cardona variety is also shown (**B3**).

Figure 1 presents scanning electron microscopy (SEM) images of mucilage extracted from the pulp (puC for the Cardona pulp and puZ for the Zacatecas pulp) and the peel (peC for the Cardona peel and peZ for the Zacatecas peel) of the red prickly pear. The mucilage from the Cardona variety exhibited a laminar structure characterized by fibers, suggesting a higher concentration of acidic polysaccharides. The mucilage from the Cardona pulp (puC) revealed a compact arrangement composed of dense layers with thick fibers on the surface. In contrast, the mucilage from the Cardona peel (peC) displayed a network of interconnected fiber layers (Figure 1(A1–A3)).

In contrast, the mucilage from the Zacatecas variety is constituted by thin layers due to neutral polysaccharides. Indeed, even due to their microstructural arrangement, the edges could be confused as arrangements of thin fibers (Figure 1(B1,B2)). Their distinct microstructure is influenced by the ratios of acidic to neutral polysaccharides, with Cardona mucilage showing a higher concentration of acidic components and Zacatecas predominantly neutral polysaccharides. These findings align with those previously reported and are consistent with the microstructure of chia mucilage [13,14]. It is essential to understand that methoxylation, methyl esterification, and the degree of methoxylation refer to the esterification of carboxylic acids (COOH) present in galacturonic acids. These processes begin with deprotonation (the loss of an H^+^) of the carboxylic acid, forming a carboxylate ion (COO^−^). The subsequent addition of a methyl group (CH_3_) leads to methoxylation and methyl esterification (COOCH_3_). Conversely, acetylation is also a type of esterification, where the carboxyl group remains in its carboxylate ion form (COO^−^). However, it loses an oxygen and gains a methyl group (CH_3_), resulting in acetylation (COCH_3_) [3,13,15].

The IR spectra (Figure 1(B3)) obtained in the range of 650–4000 cm^−1^ revealed two important regions. The first region (650–1400 cm^−1^) is considered the fingerprint of all polysaccharides, providing information about the length of their main chain [16]. The 885 cm^−1^ band corresponds to the C–H group of the aromatic rings of pyranose, galactose, and glucose, representing neutral polysaccharides in this work, and the signal at 1013 cm^−1^ is characteristic of vibrations of the α(1→4) C–O–C bond of galacturonic acids (acidic polysaccharides). This band was more intense for the mucilage obtained from the peel than that from the pulp, which is attributed to the longer main chain in the peel mucilage of the red prickly pear.

The second region (1400–1800 cm^−1^) revealed key functional groups in the mucilage, such as carboxylic acids, carboxylates, ether groups, and alcohols. The bands at 1616 cm^−1^ and 1418 cm^−1^ represent the symmetric and asymmetric stretches typical of the carboxylate ion (COO^−^) in the mucilage [17]; these bands correspond to the ionized, non-esterified form of the carboxylic group, while the bands at 1720 and 1738 cm^−1^ represent the stretches of the carbonyl group C=O of methoxylated carboxylic acids. The greater intensity of the signals at 1616 cm^−1^ and 1418 cm^−1^ compared to those at 1720 cm^−1^ and 1738 cm^−1^ suggests a low degree of methoxylation (DM) in the mucilage, which is consistent with reports for mucilage from various *Opuntia* spp. [17]. Notably, the pulp exhibited a lower DM compared to the peel. This low degree of methoxylation indicates that carboxyl groups were readily available for interaction with water molecules, explaining the significant water retention capacity of the acidic polysaccharides. These groups can also interact with other components, such as calcium ions or sugars, forming structural networks in the presence of water. The band at 2933 cm^−1^ corresponds to vibrations of CH– functional groups found in the sugars constituting the side chains of the mucilage’s chemical structure. A higher presence of these sugars was observed in the peel’s mucilage, indicating a more branched structure and, consequently, higher water retention and viscosity than pulp-derived mucilage. Additionally, the band at 3272 cm^−1^ corresponds to stretches of OH groups present in sugars and galacturonic acids, facilitating the formation of intermolecular hydrogen bonds within the mucilage structure. This interaction is crucial for the technological properties of mucilage, such as gel formation, film development, and emulsification. Unfortunately, the mucilage from the Zacatecas variety could not be analyzed using Raman spectroscopy due to fluorescence interference, which was likely linked to the presence of betalains, indicating a strong interaction between these compounds.

#### 3.2.2. Pectin

**Figure 2:**
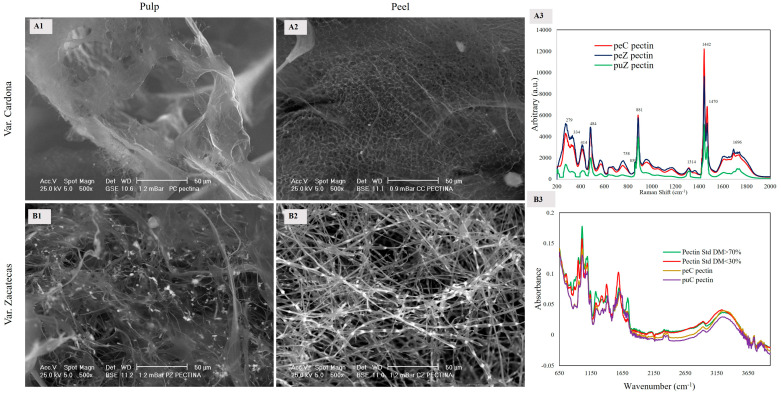
Imagen SEM images (500×) of pectin from the peel and pulp obtained from the red prickly pear, Cardona (**A1**,**A2**) and Zacatecas varieties (**B1**,**B2**). IR (**B3**) and Raman (**A3**) spectra of the pectin obtained from the pulp and peel of both varieties are also shown.

The SEM images in Figure 2 show the pectin extracted from the pulp and the peel of the two varieties of red prickly pear. Pectin is a heterogeneous and complex group of polysaccharides present in the cell walls of plants, which is primarily composed of D-galacturonic acids linked by α-1-4 bonds, with approximately 65% of these acids forming such linkages. Additionally, pectin can be esterified or unesterified, as well as methoxylated or acetylated at C_6_ [18].

Figure 2 shows the microstructure of the pectin extracted from the peel and pulp of both varieties, highlighting how its microstructure reflects its chemical composition. The pectin from the Cardona pulp (puC) exhibits a laminar and homogeneous morphology (Figure 2(A1)), which can be attributed to a higher content of neutral sugars. This composition promotes the formation of bonds between the hydroxyl groups (OH) of the neutral sugars and the carboxyl groups (COOH) of galacturonic acids [19]. Neutral polysaccharides that can be present in the main and side chains of pectin include residues of α-D-galactopyranose, α-L-arabinofuranose, and rhamnopyranose, which are linked by α-(1,2) bonds [20].

In contrast, the pectin from the pulp of the Zacatecas (puZ) variety presents a heterogeneous structure characterized by the predominance of thin fibers (Figure 2(A2)). This structural difference arises from a lower presence of neutral sugars (corroborated by Raman spectroscopy), which prevents the formation of bonds and promotes charge repulsion, thereby inhibiting the formation of homogeneous laminas (Figure 2(B1)). Regarding the peel pectin, the pectin from the Cardona variety (peC) (Figure 2(A2)) exhibits a homogeneous network-like structure with small spaces. These spaces result from water molecules prior to lyophilization [18,21], attributed to a well-balanced ratio of galacturonic acids and neutral sugars, as confirmed by the intensity of the 1401 cm^−1^ band in FTIR and 1470 cm^−1^ in Raman spectra. This homogeneous structure promotes high water retention. In the case of peZ (Figure 2(B2)), pectin also presents a network-like structure but with more defined fibers associated with bright points (calcium oxalate crystals). This phenomenon is due to the predominance of galacturonic acids, which exhibit a higher affinity for divalent cations due to their low degree of methoxylation [22]. Furthermore, Zacatecas variety pectin could have rhamnose residues, which is significant, as it has been shown to act as a steric barrier, complicating the formation of a regular and ordered “egg-box” structure and limiting chain growth in binding zones, which affects the gelation of pectin [23,24]. These results are consistent with previous studies indicating that the microstructure of pectin is dependent on the degree of methylation and the content of calcium ions [21]. All the aforementioned information regarding pectin morphology was confirmed through IR and Raman spectroscopy, which will be presented subsequently.

The 650–1500 cm^−1^ region of the FTIR spectra presents different characteristic bands corresponding to the “fingerprint” of polysaccharides. Bands in this zone correspond to complex vibrational interaction systems, and they were analyzed collectively. Inside this region, the 700–1000 cm^−1^ bands that are challenging to assign to functional group vibrations were observed. The 1000 to 1200 cm^−1^ region corresponds to vibrations of C–O and C–C bonds of the glycosidic linkage or the pyranose ring. In this region, the band at 1011 cm^−1^ is characteristic of vibrations corresponding to the α-1-4-C–O–C linkage of galacturonic acids, and its intensity is proportional to the polymerization degree [20].

In Figure 2(A3), the FTIR spectra of peC and puC pectin are presented alongside two pectin standards with different degrees of methoxylation (DM 30 and 70%). The DM > 70% pectin standard exhibits greater intensity in the 1011 cm^−1^ band, followed by the DM < 30% pectin standard. peC pectin shows higher intensity than puC (1011 cm^−1^ band), indicating higher polymerization (greater content of galacturonic acids). Between 1230 and 1401 cm^−1^ are the signals that allow the determination of the pectin methoxylation degree. The 1235 cm^−1^ band corresponds to asymmetric vibrations of the methyl group (CH_3_); its intensity is proportional to the pectin methyl group content [20], confirming the presence of methoxylated carboxylic acids. This band is more intense for the DM > 70% pectin standard, while peC and puC pectin show similar intensities to the DM < 30% standard, implying low methoxylation. The band at 1401 cm^−1^ reveals free carboxylic acids interacting with ions or amines, consistent with Raman findings, which are more intense for the DM < 30% pectin standard.

In the region between 1500 and 1800 cm^−1^, characteristic bands of carboxylic acid vibrations in the form of carboxylate ions, methoxylated or acetylated, may serve as an indicator of DM. Particularly, the band at 1630 cm^−1^ corresponds to the carboxylate ion, while the signal at 1749 cm^−1^ arises from the double bond of the carbonyl group (C=O) of protonated and methoxylated carboxylic esters [24]. In Figure 2(B3), the high methoxylation (DM > 70%) pectin standard exhibits a signal at 1630 and 1736 cm^−1^, while the low esterification (DM < 30%) pectin standard only shows the band at 1593 cm^−1^. puC and peC pectin only present the carboxylate ion signal close to 1593 cm^−1^, indicating its low degree of methoxylation. Therefore, its carboxylic groups are not esterified and exist in the form of free carboxylate ions (COO^−^). These findings are similar to reports on pectin obtained from different fruits [16,25]. Finally, the band at 3244 cm^−1^ corresponds to the stretching vibrations of hydroxyl groups, mainly from sugars present in the branching structure of pectin.

Pectin was also characterized using Raman spectroscopy. In this region, stretching modes of the α-1-4 linkages between galacturonic acids, _ν_(COC), were observed at 478 and 854 cm^−1^. The latter band reflects acetylation and/or methylation, indicating the anomeric configuration of carbohydrates. If there is a shift between 825 and 860 cm^−1^, it corresponds to equatorial anomeric hydrogen (α-anomers and α-glycosides), while if it shifts between 880 and 900 cm^−1^, it corresponds to axial anomeric hydrogen (β-anomers and β-glycosides). The band at 1442 cm^−1^ corresponds to the in-plane deformation vibrations of the methyl groups _δ_(CH_3_), which is characteristic of acetylated ester groups (COCH_3_) in low methoxyl pectin. The characteristic signal of methoxylated esters is presented at 1458 cm^−1^ [20].

Figure 2(B3) shows the Raman spectra of the pectin (pulp and peel) from two varieties. Two bands (859 and 881 cm^−1^) were identified, indicating a low degree of methoxylation, as there were no shifts or signals < 857 cm^−1^. The signal at 1470 cm^−1^ confirmed that the samples had a low degree of methoxylation. Finally, the band at 1740 cm^−1^ confirmed the presence of galacturonic acids, corresponding to the stretching of the C=O groups of the COOH carboxylic acids of galacturonic acids.

Raman, IR, and SEM spectroscopic analysis revealed the low degree of methoxylation of the red prickly pear pectin. The identification of specific functional groups indicated their interaction with calcium ions, abundant in *Opuntia* fruits and cladodes. These interactions facilitate gel formation at elevated temperatures, resulting in the characteristic egg-box structure.

#### 3.2.3. Hemicellulose

**Figure 3:**
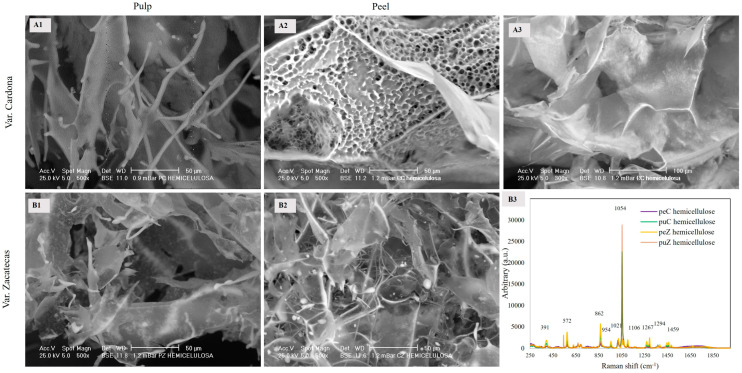
SEM images of hemicellulose extracted from the pulp ((**A1**,**B1**): 500×) and peel ((**A2**,**B2**): 500×; (**A3**): 300×) of the red prickly pear. Raman spectra of the hemicelluloses are presented in (**B3**).

Hemicellulose is another type of polysaccharide present in plants that is characterized by a high content of hydroxyl groups distributed along its main and side chains. These hydroxyl groups allow for the generation of steric or orientation repulsions between hemicellulose fibers, preventing their agglomeration [26]. Hemicellulose is a heterogeneous group of polysaccharides characterized by a β-(1→4) linkage that connects sugar molecules in an equatorial configuration [27]. Figure 3 shows SEM images of the microstructure of hemicellulose along with their respective IR spectra. The puC hemicellulose exhibits a structure composed of dense layers (Figure 3(A1)), in contrast to puZ, which consists of thin layers (Figure 3(B1)). The peC hemicellulose demonstrates greater heterogeneity, characterized by a combination of porous and smooth zones (Figure 3(A2,A3)). In contrast, the thin, fibrous layers in peZ hemicellulose suggest enhanced elasticity, a property linked to its carboxylic acid content confirmed by Raman bands at 1267 and 1459 cm⁻¹ (Figure 3(B2)), as noted in previous reports [28], compared to the porous layers of peC. Figure 3(B3) shows the Raman spectra of hemicelluloses peC, peZ, puC, and puZ. The band at 1054 cm^−1^ corresponds to the main chain signal of this polysaccharide, representing the stretching of CC and CO of β-(1-4) C–O–C glycosidic linkages and stretching vibrations _V_(CO) and OH [29]. The branching signals were found at 1459 and 1294 cm^−1^, corresponding to in-plane deformations of C–H, C–H–O, δ(COH), and the 1° and 2° alcohol groups of xylans, as well as the scissoring vibrations of long carbohydrate chains _δ_(CH_2_).

The signal at 1267 cm^−1^ corresponds to deformations in the CH_2_, HCC, HCO, and COH planes and symmetric vibrations of _V_COC, confirming the presence of glycosidic linkages, while those at 1106 and 572 cm^−1^ correspond to vibrations and other deformations in the plane related to the C–O–C linkage of the cellulose ring [21,24] related to xylan residues reported at 563 cm^−1^ [30]. The band at 391 cm^−1^ is associated with in-plane ring vibrations _δ_(CCC) of cellulose [31]. The fibers observed in the peZ morphology (Figure 3(B2)) may be associated with the higher intensity of the characteristic Raman bands corresponding to branching (1267, 1294, and 1459 cm^−1^); in contrast, the porosity observed in peC (Figure 3(A2)) is linked to the absence of these signals. Hemicellulose exhibiting a structure composed of thin layers and fibers provides greater elasticity compared with a structure of porous layers because a fibrillar structure is related to a greater presence of carboxylic acids (confirmed by Raman spectroscopy), which give it hydrophilic properties and more bonds with polar compounds, increasing its flexibility.

#### 3.2.4. Cellulose

**Figure 4:**
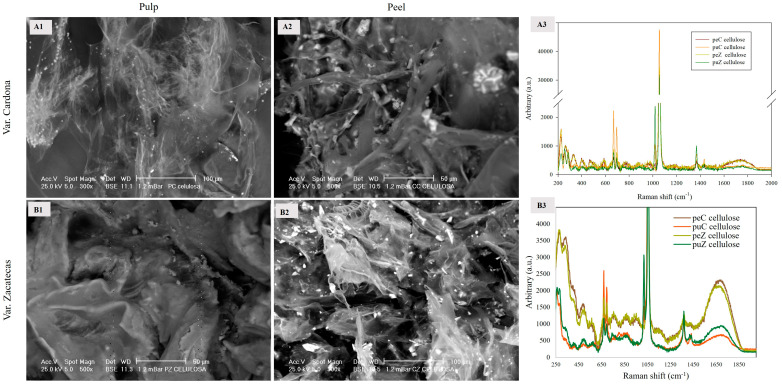
SEM images of the cellulose extracted from the pulp ((**A1**): 300×; (**B1**): 500×) and cellulose extracted from the peel ((**A2**), 500×; (**B2**), 300×) of the red prickly pear. Raman spectra of the celluloses are presented in (**A3**) and (**B3**).

Cellulose is a linear polysaccharide composed of long chains of β-D-glucopyranose linked by glycosidic bonds between carbon 1 and 4 (β, 1→4), which are associated with bundles connected by hydrogen bonds [27]. Figure 4 shows the SEM images of the cellulose microstructure obtained from the puC, puZ, peC, and peZ, as well as their respective Raman spectra.

Cellulose extracted from the peel (Figure 4(A2,B2)) exhibited calcium crystal, contributing to its rigidity, as opposed to the smoother morphology of pulp cellulose (Figure 4(A1,B1)). Specifically, puC cellulose (Figure 4(A1)) exhibited thin and/or delicate layers with thin fibers, while puZ comprised dense layers (B1). The peC morphology (Figure 4(A1)) consisted of dense layers with bundles of fibers, like those reported previously [31,32], providing it with greater rigidity; meanwhile, peZ (Figure 4(B2)) is composed of thin and rigid layers formed by thin fibers [11].

Figure 4(A3) corresponds to the Raman spectra of cellulose extracted from peC, puC, peZ, and puZ. The most significant signal appeared at 1054 cm^−1^, characteristic of the asymmetric stretching of β-(1-4) C–O–C glycosidic linkages and polarization-dependent changes, with puC cellulose standing out due to its intensity. The orientation sensitivity of this band can be used to prove changes in the cellulose microfibril orientation [11].

Figure 4(B3) shows the amplification of the weaker signals. At 668 and 693 cm^−1^, stretching in the _δ_(C–C) plane of the rings characteristic of cellulose was observed, with greater intensity found for the Cardona variety. At 1018 cm^−1^, the signal corresponding to CC and CO stretching of cellulose was detected, and the signals at 1375 and 1433 cm^−1^ correspond to symmetric and asymmetric in-plane deformations of the methyl groups _δ_(CH_3_) [31,33,34,35]. The differences in the characteristic bands presented in the Raman spectra, such as 1018 and 1375 cm^−1^, as well as the presence of calcium crystals, confer microstructural differences between the cellulose from the pulp and that from the peel, as confirmed by SEM images. All FTIR and Raman spectral bands discussed in this manuscript are detailed in Table 2.

## 4. Conclusions

The extraction and purification of the four main polysaccharides present in the pulp and peel of the red prickly pear fruit were successfully achieved using environmentally friendly solvents. This highlights the red prickly pear as a good source of polysaccharides.

This study established a correlation between the functional groups of each of the polysaccharides, an important aspect of their molecular structure, and their microstructural properties. SEM and spectroscopy analyses proved to be effective techniques for evaluating their chemical composition and microstructure. These findings enhance the potential of these polysaccharides as eco-friendly ingredients in food formulations and sustainable materials for packaging.

SEM imaging provided valuable insights into the spatial arrangements and microstructural features of the polysaccharides, including mucilage, pectin, cellulose, and hemicellulose. These images revealed characteristics such as fiber alignment, porosity, and layer formation. Meanwhile, FTIR and Raman spectroscopy identified key functional groups, including hydroxyl, carboxyl, and methyl groups, which are critical for understanding the chemical properties of the polysaccharides.

This paper contributes to establishing the correlation between microstructural characteristics observed by SEM and chemical characteristics obtained by FTIR and Raman spectroscopies of red prickly pear polysaccharides, by employing innovative, non-invasive techniques like microscopy and spectroscopy. Understanding the chemical composition of polysaccharides and how it influences their microstructural characteristics provides the foundation for elucidating the interactions of these compounds with other components in a food matrix, enabling the development of foods with specific characteristics.

## Figures and Tables

**Table 1:** The polysaccharide content in the pulp and peel of two varieties of the red prickly pear (percentages of dry weight).

Variety	Part of the Fruit	Cellulose (%)	Hemicellulose (%)	Pectin (%)	Mucilage (%)
Cardona	Pulp	12.65 ± 0.58 ^d^	3.060 ± 0.83 ^c^	0.21 ± 0.041 ^a^	1.98 ± 0.43 ^b^
	Peel	46.81 ± 0.47 ^d^	9.19 ± 0.25 ^b^	19.20 ± 0.16 ^c^	4.45 ± 0.29 ^a^
Zacatecas	Pulp	21.92 ± 0.64 ^d^	2.42 ± 0.23 ^b^	14.14 ± 0.72 ^c^	0.95 ± 0.12 ^a^
	Peel	55.53 ± 0.14 ^d^	0.99 ± 0.09 ^a^	19.52 ± 0.09 ^c^	5.17 ± 0.65 ^b^

Different letters indicate statistically significant differences (*p* ≤ 0.05).

**Table 2:** A summary of the Raman and IR bands assignments for red prickly pear polysaccharides.

Raman Shift (cm^−1^)	IR Shift (cm^−1^)	Assignments	Polysaccharides	References
3550–2700		Stretching Methyne (R–CH=R) Methylene (R–CH_2_–R)	Cellulose	[29]
	3272	OH, C–H, N–H, CH_2_	Mucilage, Neutral	[36,37]
	3244	OH	Pectin	[38]
	2940	Alkanes stretching (sugars)	Pectin	[38]
	2933	C–H Alkanes	Mucilage, Neutral	[37]
	2376	C–H Alkanes	Mucilage, Neutral	[37]
	2307	C–H Alkanes	Mucilage, Neutral	[37]
1750–800		Methyne bending (R–CH=R) Methylene wagging and rocking (R–CH_2_–R)	Cellulose	[29]
	1780	Stretching COOH	Pectin	[24]
	1749	Carboxylic ester groups (COOCH_3_)	Pectin	[24]
1740		Carbonyl group (C=O) of COOH	Pectin	[9,20]
	1736	Protonated carboxylic ester groups (COOCH_3_)	Pectin	[24]
	1720	C=O	Mucilage, Acid	[37]
	1630	Carbonyl group C=O	Pectin	[24]
	1616	Carbonyl (C=O) and NH2	Mucilage, Acid	[36,37]
	1593	Carboxylate ion (COO–)	Pectin	[24]
	1540	COOH stretching	Mucilage, Pectin	[24]
1470		COCH_3_ (acetylation)	Pectin	[9,20]
1459		C–H and C–H–O, δ(COH), δ(CH_2_)	Hemicellulose	[31]
1442		Methyl groups (CH_3_)	Pectin	[9,20]
1433		Symmetric and asymmetric stretching of methyl groups (δCH_3_)	Cellulose	[29,31]
	1418	COH	Mucilage, Acid	[37]
1375		Symmetric and asymmetric stretching methyl groups (δCH_3_)	Cellulose	[31]
1294		C–H and C–H–O, δ(COH), δ(CH_2_)	Hemicellulose	[31]
1267		CH_2_, HCC, HCO, COH, and COC glycosidic linkage	Hemicellulose	[31]
	1234	C–O	Mucilage, Acid	[37]
	1230	Methyl (CH_3_)	Pectin	[20]
1106		C–O–C of the ring	Hemicellulose	[29,31]
1054	1097	β-(1-4) C–O–C linkage	Hemicellulose, Cellulose	[11,29,31]
1018		CC, CO	Cellulose	[29,31]
	1013	α-1-4-C–O–C bond	Mucilage, Acid	[37,39]
	1011	α-1-4-C–O–C linkage	Pectin	[20]
	885	C–H	Mucilage, Neutral	[36,40]
881		Acetylated COOH groups (COCH_3_)	Pectin	[20]
859		Acetylated COOH groups (COCH_3_)	Pectin	[20]
693		(δ C–C) glucose benzene	Cellulose	[29]
668		(δ C–C) glucose benzene	Cellulose	[29]
610		CCC, COC, OCC, and OCO	Cellulose	[29]
577		δ CCO and δ CCC of the cellulose ring	Cellulose	[31]

## Data Availability

The original contributions presented in the study are included in the article, further inquiries can be directed to the corresponding author.
